# Cervicovaginal microbiome modulates HPV genotype-specific clearance/persistence: role of bacterial dysbiosis in cervical carcinogenesis

**DOI:** 10.1080/07853890.2026.2754577

**Published:** 2026-11-06

**Authors:** Jiahai Zhang, Mingyu Ji, Nan Lv, Yunying Zhou

**Affiliations:** Medical Research & Laboratory Diagnostic Center, Central Hospital Affiliated to Shandong First Medical University, Jinan, China

**Keywords:** Cervicovaginal microbiome (CVM), HPV 16/18 persistence, HPV 52/56/58 clearance, 16S rRNA sequencing, host-microbe interaction

## Abstract

**Objectives:**

This study focuses on the distinct epidemiological features of high-risk human papillomavirus (hrHPV) genotypes in Asian populations: HPV52/56/58 show uniquely high prevalence and strong correlation with cervical intraepithelial neoplasia, but their detection rates drop sharply in invasive cervical cancer, a pattern clearly different from the persistent dominance of HPV16/18 throughout malignant progression.

**Methods:**

To further elucidate the potential host-microbe interactions underlying these epidemiological observations, we integrated a 16S rRNA-sequenced cross-sectional discovery cohort and an independent longitudinal validation cohort from a Chinese population, with strict experimental quality control throughout the workflow.

**Result:**

The preliminary analytical results demonstrate that a *Lactobacillus*-dominant cervicovaginal microbiome (CVM) is significantly and positively associated with HPV viral clearance, while enriched abundance of *Gardnerella*, *Saccharofermentans* and *Atopobium* exhibits a robust correlation with persistent HPV52/56/58 infection. Three undercharacterized microbial taxa-*Chryseobacterium*, *Sphingobacterium* and *Enhydrobacter* are closely linked to sustained HPV16/18 prevalence across advanced disease stages, and show negative correlation with the above obligate anaerobes, which aligns with the sharp decline of HPV52/56/58 in tumor tissues. A hormone-linked *Chryseobacterium* enrichment were also identified, a taxon with previously reported progesterone-degrading functional properties.

**Conclusion:**

While further longitudinal studies are required to establish causal relationships, our results indicate that CVM dysbiosis may modulate the natural history of these high-burden HPV genotypes, and provide a foundational rationale for subsequent development of genotype-specific, microbiota-targeted cervical disease intervention strategies.

## Introduction

Epidemiological investigations have disclosed that approximately 80% of women worldwide experience human papillomavirus (HPV) infection during their lifetime [1,2]. The transition from HPV infection to cervical cancer is a protracted and intricate process, typically spanning 5–10 years. During this period, the status of cervical intraepithelial neoplasia can either regress, remain stable, or ultimately progress to cervical cancer [3,4]. The prevalence of HPV exhibits significant variation across different regions, nationalities, and environmental settings [5,6]. HPV16 and 18 have long been recognized as the most oncogenic genotypes. In China and across Asia, HPV genotypes 52 and 58 rank as the second and third most prevalent infections, respectively. Notably, numerous studies have demonstrated a strong association between HPV 52 and 58 and the development of Cervical Intraepithelial Neoplasia (CIN) [3,4,7]. Paradoxically, their detection rates decline in cases of cervical cancer. Regrettably, the underlying mechanisms governing the clearance of HPV 52/56/58 and the persistence of HPV 16/18 in cervical cancer, potentially involving genetic, immunological, and metabolic factors, remain poorly understood.

Accumulating evidence indicates that the transformation of normal cells into malignant ones is a multifactorial process, strongly influenced by dysregulation of the microbiome. Notably, HPV infection alone is insufficient to trigger cervical cancer [8–11]. The cervicovaginal microbiome (CVM) represents a dynamic ecosystem, and its community state types (CSTs) are characterized by specific bacterial consortia that establish symbiotic relationships within the CVM [12–15]. *Lactobacillus*, the dominant bacterium in the CVM, plays a pivotal role in maintaining the vaginal microecological balance. Higher frequencies of certain CSTs have been linked to increased disease severity [16]. Recent clinical studies have increasingly recognized chronic inflammation as a ‘driver’ of carcinogenesis. For instance, vaginal lavage [17,18], bacterial vaginosis (BV) [19], and sexually transmitted infections [20,21] can disrupt the vaginal microenvironment, thereby facilitating the persistence of HPV infection. Moreover, the CVM is not only characterized by symbiotic relationships but also by antagonistic interactions. The CVM has emerged as a potential biomarker for the progression and regression of HR-HPV infections [16,22,23], b although its relationship with the severity of cervical neoplasia remains controversial [24,25]. Emerging evidence suggests that the composition of the anaerobic vaginal microbiome is associated with a reduced rate of HPV regression, with *Gardnerella*’s effects potentially mediated by CVM diversity [17–19]. However, the role of other microbial species in the regression, persistence, or progression of cervical lesions, as well as their impact on the clearance and persistence of different HPV genotypes, remains to be elucidated.

Previous studies have extensively explored the relationship between CVM and HR-HPV infection, identifying distinct microbial signatures across different stages of cervical health and dysplasia. However, the majority of these studies have focused on HPV16/18 genotypes [20], overlooking the genotype-specific infection patterns in different populations during cervical carcinogenesis. Additionally, several studies have suggested that an imbalance between estrogen and progesterone may contribute to the development of cervical cancer [21]. Nevertheless, the precise nature of the relationship between estrogen and progesterone levels and cervical cancer remains unclear. It is thus of interest to determine whether specific components of the vaginal flora can modulate the levels of these hormones and influence the progression of cervical cancer. In this study, we aimed to investigate the association between CVM and the clearance/persistence of different HPV genotypes in the Chinese population, thereby providing novel insights into the potential regression of cervical lesions or differential responses to treatment.

## Materials and methods

### PubMed database search

We systematically retrieved published studies from PubMed, and aggregated data from a total of 1.7 million Chinese female participants across these eligible studies, covering 25 cities of China (Figure S1). The 25 cities are Beijing, Xinjiang, Liaoning, Hebei, Shandong, Shanxi, Ningxia, Gansu, Shaanxi, Henan, Jiangsu, Anhui, Hubei, Sichuan, Zhejiang, Jiangxi, Hunan, Guangzhou, Fujian, Yunan, Guangxi, Guangdong, Taiwan, Tibet, and Macao.

### Participant selection procedure

#### Population foundation

The source population of this study consists of 24,641 women aged 21–70 years who underwent HPV genotyping testing at the Gynecology Outpatient Clinic and Health Check-up Center of Jinan Central Hospital Affiliated to Shandong First Medical University from January 2018 to December 2023 (Table S1). This large cohort is explicitly defined as the sampling basis for all subsequent sub-cohorts.

#### Preliminary screening stage

From the above source population with documented HPV genotyping results, women who completed cervical disease diagnosis, treatment, and subsequent follow-up in our hospital were selected as the preliminary screening pool for the 16S rRNA sequencing cohort.

#### Exclusive screening for longitudinal cohort

The longitudinal follow-up cohort restricted enrollment to women aged 33–70 years with high-grade squamous intraepithelial lesion (HSIL) and cervical cancer, covering the perimenopausal to early postmenopausal population to match the population characteristics required for subsequent hormone-related analyses. The cohort was asked to participate in a 1-year follow-up visit for repeated HPV testing and RT-PCR verification.

#### Unified exclusion criteria

For the HPV-negative control subgroup, individuals with any prior documented HPV infection history or HPV vaccination record were excluded. For all participants in the HPV-positive group, the following exclusion criteria were applied: current pregnancy, within 8 weeks postpartum, sexual intercourse or vaginal douching within 3 days prior to sampling, ongoing use of specified medications or receipt of local vaginal interventions, prior hysterectomy, previous ablative destructive therapy on the cervix, personal history of cervical malignancy or other lower genital tract cancers, and any documented history of HPV vaccination.

### Grouping information and sample size estimation

360 specimens were divided into three groups: ➀ Experimental group of 228 specimens for 16S rRNA sequencing to explore statistically significant different species: HPV negative control (NC, *n* = 20); HPV positive group (HPV16, *n* = 21; HPV18, *n* = 14; HPV52, *n* = 29; HPV56, *n* = 18; HPV58, *n* = 24; other HPV types, *n* = 81; Tumor group (TG, *n* = 21). ➁ Validation group of 60 specimens for RT-PCR detection to verify the result of 16S RNA sequencing: HPV negative control (NC, *n* = 10); HPV positive group (HPV, *n* = 20); Low-grade Squamous Intraepithelial Lesion group (ISIL, *n* = 10); High-grade Squamous Intraepithelial Lesion group (HSIL, *n* = 10); Tumor group (TG, *n* = 10). 60 additional samples and a longitudinal cohort of samples before and after tumor resection (HSIL, 6 pairs; TG, 7 pairs) for validation study (Table 1). In addition, 46 serum samples were used for hormone detection, 21 of which were premenopausal samples with cervical cancer and 25 of which were postmenopausal samples with cervical cancer, aged 54–69 years (Figure S2). The sample size of each sub-cohort for 16S rRNA sequencing in this study was determined based on the effect size of differential species between groups obtained from our preliminary experiment. With a two-sided significance level α of 0.05 and statistical power (1-β) of 0.8, the calculated minimum required sample size for each subgroup is no less than 12 cases. Our actual enrolled sample size is far above this threshold, which fully guarantees sufficient statistical power to identify differentially abundant bacterial taxa across all included groups. The 60 validation specimens for RT-PCR detection and 13 pairs of longitudinal follow-up paired samples were determined according to the specimen reserve capacity of the clinical biobank in our center, combined with the minimum sample size requirement for qPCR technical validation.

**Table 1.** The information of longitudinal samples taken from surgery and reexamination.

			HPV genotypes		Reexamination time	
	Pathological stage	Age(year)	Tumor specimen	Reexamination specimen	(Months)	Operation type
1	CIN II	49	HPV 16+, 31+	HPV-	3	Conization
2	CIN III	49	HPV 16+	HPV 58+	3	Conization
3	CIN III	37	HPV 16+, 56+	HPV 44+	3	Conization
4	CIN III	33	HPV 16+, 52+	HPV 52+	3	Conization
5	CIN III	43	HPV 16+, 52+	HPV-	3	Conization
6	CIN III	56	HPV 16+	HPV-	3	Conization
7	Superficial infiltrating squamous cell carcinoma	37	HPV 16+	HPV-	3	Conization
8	CCIA1	56	HPV 16+	HPV-	3	Radical trachelectomy
9	CCIA2	39	HPV 16+	HPV-	3	Radical trachelectomy
10	CCIB1	52	HPV 16+. 18+	HPV-	3	Radical trachelectomy
11	CCIB2	43	HPV 16+. 52+, 33	HPV-	3	Radical trachelectomy
12	CCIB	47	HPV 16+. 58+	HPV-	3	Radical trachelectomy
13	CCIIA1	70	HPV 16+	HPV-	3	Radical trachelectomy

### Sampling procedure

Cervical samples were collected using sterile cotton swabs and placed in sterile containers. Samples were immediately frozen and stored at −80 °C until they were utilized. Microbial DNA was extracted from each sample using a DNA extraction kit (Qiagen, Hilden, Germany), following the manufacturer’s instructions. Microbial DNA extracts collected above were kept at −20 °C.

### HPV assay and genotyping

HPV genotyping was performed using Sinochips LINEAR ARRAY^®^ HPV Genotyping Test to detect 18 high-risk HPV genotypes (HPV-16, −18, −26, −31, −33, −35, −39, −45, −51, −52, −53, −56, −58, −59, −66, −68, −73, −82) and 6 low-risk HPV genotypes (HPV-6, −11, −42, −43, −44, −84).

### Real-time PCR assays

Twelve DNA sequences targeting 12 microorganisms (*Lactobacillus genus* [8], *Bacteroides* genus, *Burkholderia* genus, *Gardnerella* vaginalis [8], *Acinetobacter lwoffii*, *Veillonella* genus, *Atopobium vaginae*, *Mycoplasma hominis*, *Enhydrobacter* genus, *Saccharofermentans* genus, *Sphingobacterium* genus, *Chryseobacterium* genus) and an internal control gene Human albumin gene were analyzed by qPCR. Primers of the *Saccharofermentans* genus (4 species), *Sphingobacterium* genus (10 species), and *Chryseobacterium* genus (20 species) were designed based on available 16S sequence data from PUBMED. The relevant species information is listed in Table 2, and the sequences of the gene probes and primers used for the real-time PCR assays are summarized in Table 3.

**Table 2.** Bacterial strains used in this study and their accession numbers in the databases.

Genus	Species
*Saccharofermentans*	*Acetigenes (NR_115340.1), Proteiniphilum strain M3/6 (NR_148807.1), Alkalibacter strain Z-79820 (NR_042834.1), Candidatus Caldatribacterium (APCU01000185.1)*
*Sphingobacterium*	*Composti (AB244764.1), Kyonggiense strain 2-1-2 (NR_108742.1), Hotanense strain XH4 (NR_108440.1), Lactis strain WCC 4512 (NR_108488.2), Alimentarium strain WCC 4521 (NR_108489.1), Cladoniae strain No.6 (NR_108441.1), Corticis strain 23D10-4-9 (NR_158090.1),Cellulitidis strain LMG 28764 (NR_156919.1), Jejuense strain GJ30-7 (NR_152666.1), Populi strain 7Y-4 (NR_151970.1)*
*Chryseobacterium*	*FRA20P152 (MG860480.1), FRA10P398 (MG860402.1), CF34P5A (MG860117.1), Nakagawai (NR_126257.1), Rhizosphaerae strain RSB3-1 (NR_125812.1), Lactis strain KC1864 (JX100821.1), Hispalense DSM 25574 strain AG13 (NR_116277.1), Lathyri strain RBA2-6 (NR_115853.1), Hagamense strain RHA2-9 (NR_115852.1), Elymi strain RHA3-1 (NR_115851.1), Frigidum strain D07 (NR_148657.1), Zeae strain JM-1085 (NR_133728.1), Aahli strain T68 (NR_133722.1), Taihuense strain THMBM1 (JQ283114.1), Oleae strain CT348 (NR_134002.1), Contaminans strain C-26 (NR_133725.1), Palustre DSM 21579 strain 3A10 (NR_116286.1), Flavum strain CW-E2 (NR_115957.1), Anthropi strain NF 1366 (NR_115099.1), Jejuense strain JS17-8 (NR_044300.1), Ureilyticum strain F-Fue-04IIIaaaa (NR_042503.1), Indologenes strain LMG 8337 (AY468450.1)*

**Table 3.** DNA Targets according to gen bank and nucleotide sequences of primers and probes used for quantitative real time PCR.

PCR target,	Sequence		
primer or probe	(5′→ 3′)	DNA target	
*Saccharofermentans genus*			81bp
Forward	TAGATACCCTGGTAGTCCAC	16S rRNA	
Reverse	ACGAGCTGACGACAACCAT	…	
Probe	FAM-TGAAACTCAAAGGAATTGACGGGGGC-BHQ	…	
*Sphingobacterium genus*			84bp
Forward	GTTGGATTCGCTAGTAATCG	16S rRNA	
Reverse	CCAGCTTTCATGGCTTGAC		
Probe	FAM-CGGTGAATACGTTCCCGGGCCTT-BHQ		
*Chryseobacterium genus*			230bp
Forward	AACTGACGCTGAGGGACGAA	16S rRNA	
Reverse	CCAAAACGAGTTAGCATCGTTTAC	…	
Probe	FAM-CGTGGACTACCAGGGTATCTAATCC-BHQ	…	
*Human albumin*			140bp
Forward	GCTGTCATCTCTTGTGGGCTGT	Exon 12	
Reverse	AAACTCATGGGAGCTGCTGGTTC	…	
Probe	FAM-CCTGTCATGCCCACACAAATCTCTCC-BHQ	…	

### 16S rDNA gene amplification and IlluminaMiSeq PE250 sequencing

Partial 16S rDNA gene sequences were amplified from the extracted DNA using the primer pair F341F (5′-ACTCCTACGGGRSGCAGCAG-3′) and R806R (5′-GGACTACVVGGGTATCTAATC-3′), which target the V3–V4 region of the 16S rDNA gene sequence. The KAPA HiFi HotStart ReadyMix PCR kit (Kapa Biosystems, Inc., Basel, Switzerland) was used for high-fidelity amplification. PCR reactions were performed in a 30 µL mixture containing 15 µL of 2 × KAPA Library Amplification Ready Mix, 1 µL of each primer (10 µM), 50 ng of template DNA and ddH_2_O. According to the manufacturer’s instructions, amplicons were extracted from 2% agarose gels, purified using the Axy Prep DNA Gel Extraction Kit (Axygen Biosciences, Union City, CA, U.S.), and quantified using Qubit^®^2.0 (Invitrogen, U.S.). All quantified amplicons were pooled to equalize their concentrations for sequencing using the Illumina MiSeq platform (Illumina, Inc., CA, USA). Paired-end reads of 250 bp were overlapped on their three ends for concatenation into the original longer tags using PANDAseq (https://github.com/neufeld/pandaseq, version 2.9). After the library was prepared, these tags were sequenced for paired-end reads of 250 bp on the MiSeq platform (Illumina, Inc., CA, USA) and then overlapped on their 3′ ends for concatenation into original longer tags. Library construction and sequencing were performed at RealBio Genomics Institute (Shanghai, China).

### Processing of sequencing data

PANDA-seq was used to assemble overlapping paired-end reads. The tags were trimmed from the barcodes, and primers were further checked for their rest lengths and average base quality. The 16S tags were restricted between 220 and 500 bp, such that the average Phred score of bases was no worse than 20 (Q20), and there were no more than 3 ambiguous N. The tag copy number was enumerated, and the redundancy of repeated tags was removed. Only tags with a frequency > 1, which tended to be more reliable, were clustered into OTUs, each of which had a representative tag. Operational Taxonomic Units (OTUs) were clustered with 97% similarity using UPARSE (http://drive5.com/uparse/), and chimeric sequences were identified and removed using Userach (version 7.0; Edgar, 2013). To minimize deviations caused by size differences, random leveling was performed for all samples with a subsample of 29,623 reads when there was sufficient sequencing depth. The assignment of bacterial taxonomy was performed against the Ribosomal Database Project (RDP) database using the RDP Classifier (http://rdp.cme.msu.edu), with a confidence threshold of 0.8. Rarefaction curves were generated using QIIME to determine sequencing depth. Shannon and Simpson indices were used to estimate the α diversity, which represented the abundance of species in a single sample, and were compared between groups with the Wilcoxon and Kruskal-Wallis rank sum tests using R3.1.0. The communities were compared based on phylogenetic distances using the weighted UniFrac metric to represent β diversity. Principal coordinate analysis (PCoA) was performed on the resulting matrix of distances between the pairs of samples. A heat map was generated. Based on the UniFrac phylogenetic distances, a significance test for the clustering of samples was performed using a one-way analysis of similarities (ANOSIM). LEfSe analysis was used to identify the abundant bacterial taxa among the groups. Only taxa that obtained a log-linear discriminant analysis (LDA) score > 2 were ultimately considered.

### 16S function prediction

The Phylogenetic Investigation of Communities by Reconstruction of Unobserved States (PICRUSt, https://picrust.github.io/picrust/citing_picrust.html) was used to study community system evolution through recessive state reconstruction. The software predicted functional composition based on 16S rDNA and a reference sequence database, these results are presented as inferred, hypothesis-generating observations rather than direct measurements of gene content or functional activity.

### Network analysis

SparCC was used to construct an association network. This information can be used to explain the mechanism by which phenotypic differences form between samples and to analyze the correlation between species abundance and metabolic pathways or other functions. Cytoscape was used to create a network structure diagram to visualize the correlation between species or metabolic function classifications and to find important patterns or other information. Only significant correlations with an adjusted *p* < 0.1 were presented in the network, which was visualized using the Cytoscape 3.0.2 software platform (http://www.cytoscape.org/).

### Random forest predictive model construction

For the Random Forest-ROC, all the differential species between groups were adopted for analysis. Random Forest package in R language is used to model the species characteristics first, determine the importance of the species characteristics, and then cross-verify the characteristics of important species. The number of features with the lowest error rate is determined to generate a new species feature table. All the samples were divided into the training set and the test set according to the ratio of 7:3, and then the training set data was used to train the model again, and the test set data was used to predict the effect of the model, and the AUC value was output. AUC > 0.8, indicating that the classification effect was good. The final model performance was independently validated in two separate validation cohorts: 60 samples covering the entire process of cervical lesions and 13 pairs of samples before and after tumor resection, to confirm the robustness of classification results.

### Estrogen and progesterone analysis

A 3–5 ml venous blood sample collected from 8:00 to 9:00 in the morning from premenopausal patients was obtained 3–5 days before the start of the next expected menstrual cycle, which reflects elevated plasma levels of estrogen and maximal plasma levels of progesterone. The collected blood was sent for determination of estrogen and progesterone levels.

### Statistical analysis

In addition to the statistical analyses mentioned above, statistical analyses of quantitative data were conducted using the Student’s t-test (SPSS 16.0, IBM, Armonk, NY, USA). Statistical significance was defined as a *p* < 0.05.

## Results

### Study design

This study aimed to explore the interplay between cervicovaginal microbiota composition, HPV genotype-specific clearance/persistence, and cervical lesion progression, with all research objectives hierarchically mapped to the three sequential experimental stages illustrated in the flowchart (Figure S1). (1) Screening Stage: We first mapped the national HPV prevalence landscape across Chinese population based on 1.7 million female samples retrieved from PubMed, and subsequently enrolled 24641 local participants for standardized HPV genotyping to clarify the regional distribution characteristics of HPV subtypes. (2) Identification Stage: 228 representative specimens were selected and subjected to high-throughput sequencing targeting the V3–V4 region of 16S rRNA gene, to screen differential bacterial taxa significantly associated with persistent HPV16/18 infection and spontaneous clearance of HPV52/56/58. (3) Validation Stage: qRT-PCR was applied to verify the relative abundance of differential taxa and confirm sequencing reliability. Paired pre- and post-tumor resection samples were used to explore peri-tumor microecological changes. Integrative analysis of serum hormones and cervicovaginal microbiota across menopausal status-stratified cohorts was conducted to clarify their association.

### General characteristics of the study population

As shown in Figure S1, the HPV genotypes were not the same in each province, and the top 5 HPV subtypes in the region where this study was conducted are 16, 52, 56, 58 and 18. The positive rates of HPV16 and 18 only accounted for 3.8% of the HPV genotype in the 24,641 specimens (Table S1). Even when considering the co-infection rate with other genotypes, the proportion increased by 1.9%, reaching 5.7%, whereas 16.8% of people infected with HPV had other genotypes. In addition, among the 16.8%, HPV52, 56, and 58 were enriched and more prevalent than HPV 18. During cervical carcinogenesis, the persistence of HPV16 or 18 in cervical lesions was enhanced, while HPV 52, 56, and 58 were increased with the severity of CIN and decreased in cervical cancer. However, the proportion of co-infection of HPV18 with HPV16 gradually increased, whereas the co-infection of HPV 16 or 18 with HPV 52, 56, and 58 gradually decreased **(**Table S2). This raised the question of whether some specific microbiota spp. are associated with the stage-specific distribution of different HPV genotypes across lesion progression.

16S rRNA sequencing of 228 samples was performed over 6 months, including 18 different HPV genotypes with the highest infection rates (Figure 1a). Among the 228 subjects finally included in this study, there are 20 cases in the HPV negative control group, 187 cases in the HPV infection group with different grades of cervical lesions, and 21 tumor patients. Among the high-risk HPV infected population, HPV16, HPV58 and HPV52 genotypes account for the highest proportions, which are 38.1%, 21.7% and 15.2% respectively. In the stratification of cervical lesions, there are 89 cases of chronic cervicitis, 58 cases of low-grade squamous intraepithelial lesion (LSIL), 40 cases of high-grade squamous intraepithelial lesion (HSIL) and 21 cases of cervical cancer. The age range of the study population is 21–70 years, with an average age of (43.7 ± 11.2) years, including 167 premenopausal subjects and 61 postmenopausal subjects. There is no statistically significant difference in age distribution among all groups (*p* > 0.05).

**Figure 1.**
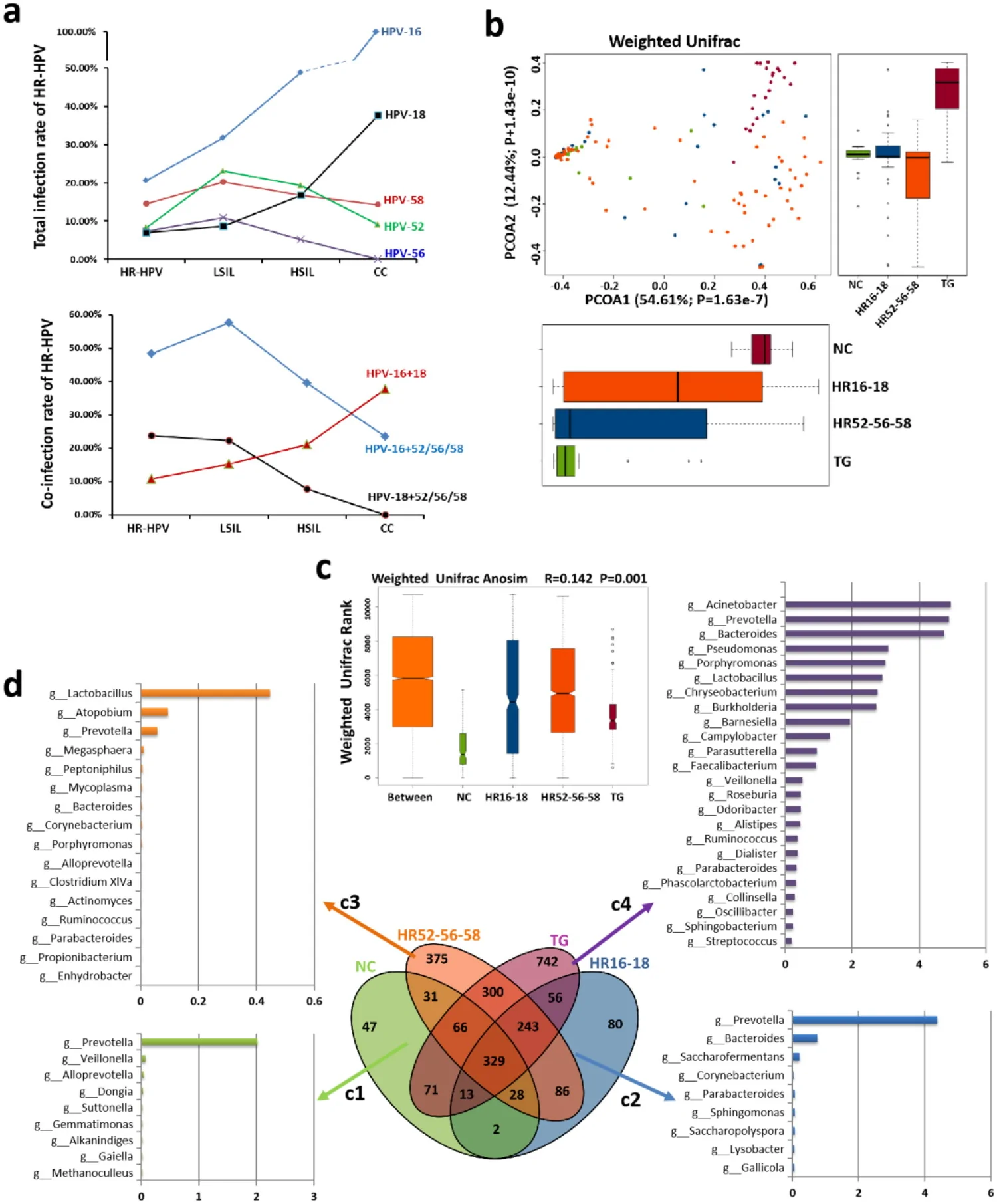
HPV prevalence characteristics and cervicovaginal microbiome features. (a) The profile of HPV single genotype infection and multiple genotype infection in the progression of cervical lesion development. (b) Significant differences were shown between groups by ANOSIM analysis (PANOSIM < 0.001). (c) Principal component analysis (PCA) with Bray-Curtis dissimilarity based on genera between the macrobiotics of groups. Points represent samples. Samples that are more similar to one another are put closer together and form distinct clusters. (d) CVM evolution during different stages of cervical cancer. Operational Taxonomic Units (OTUs) Venn diagram analysis, which can well reflect the number of OTUs shared between groups and unique within groups by using R (v3.5.1) language.

**Figure 2.**
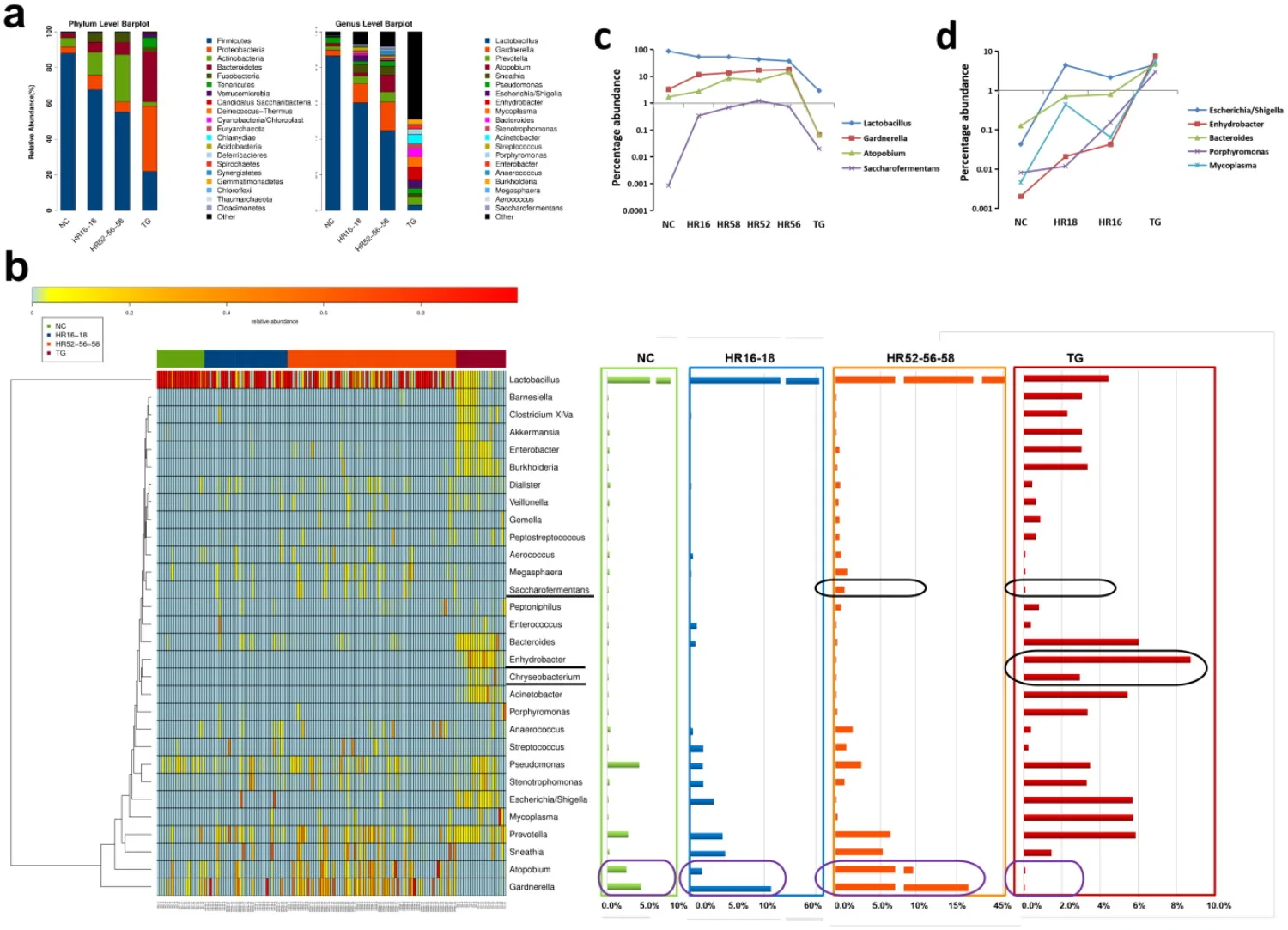
The overall structure of the cervicovaginal communities. (a) The difference in the abundance of cervicovaginal microbiome (CVM) communities at the Phylum and Genus levels. (b) The top 30 genera with significant richness difference (*p* < 0.05) between the groups. The genera circled on the right side were associated with HPV clearance and persistence, while the genera underlined on the left side were newly found in this study. (c) The existence of similar bacteria in the vagina contributes to HPV16, 52, 56, and 58 co-infection. (d) The existence of similar bacteria in the vagina contributing to HPV16 and 18 co-infection.

Through cluster analysis of OTUs, the total and specific numbers of OTUs were found to be positively correlated with the infection rate of HPV genotypes, whereas the species diversity and specific species number of the HPV16, 52, 56, and 58 infection groups were more similar than those of HPV18 (Figures S3–S4). This is consistent with the co-infection data described, which may explain why HPV52, 56, and 58 are more likely to be co-infected with HPV16 than with HPV18. To clarify the persistence of HPV16 and 18 and the clearance of HPV52, 56, and 58 with the progression of cervical lesions, we combined the five individual groups into HR16-18 and HR52-58-56 groups. α-diversity, β-diversity, and MRPP analysis indicating the grouping had statistical significance (Figure 1b, Figure S5). The principal component analysis (PCoA) analysis showed that with the development of cervical lesions, there were some similar microbiota compositions between the two adjacent groups, but they also tended to separate from each other, which was consistent with the characteristics of prolonged time and reversibility of the development of cervical lesions (Figure 1c). Figure 1d suggests that the species diversity and number of unique species of the HR52-56-58 infection group were significantly higher than those of the HR16-18 group.

**Figure 3.**
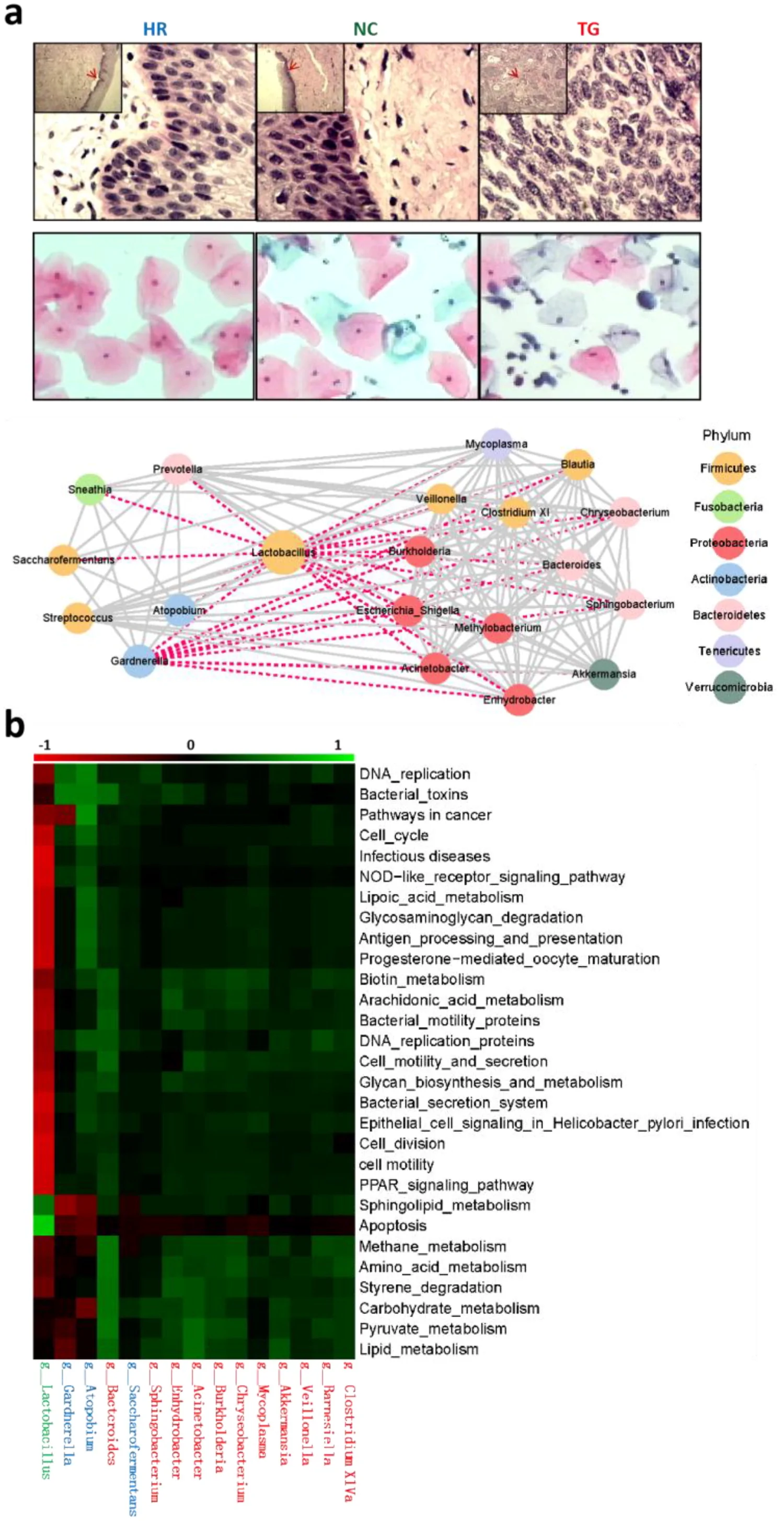
Functional profiling of CVM associated with persistence vs. clearance. (a) SparCC network plot of co-abundance and co-exclusion correlations between differentially abundant bacteria and opportunistic pathogens. Each node represents one species, and two nodes are linked if the correlation was significant, and this figure shows the network of significant correlation for the top 80. Lines between nodes show positive correlations (solid lines) or negative correlations (dashed lines). The node size is proportional to the mean relative abundance of species in the enriched population. (b) Microbial functions predicted using The Phylogenetic Investigation of Communities by Reconstruction of Unobserved States (PICRUSt) based on the closed-reference OTUs at the third level of the Kyoto Encyclopedia of Genes and Genomes (KEGG) pathway. KEGG pathways with significant abundance differences (*p* < 0.05) are shown. Green represents a positive correlation, and red represents a negative correlation.

**Figure 4.**
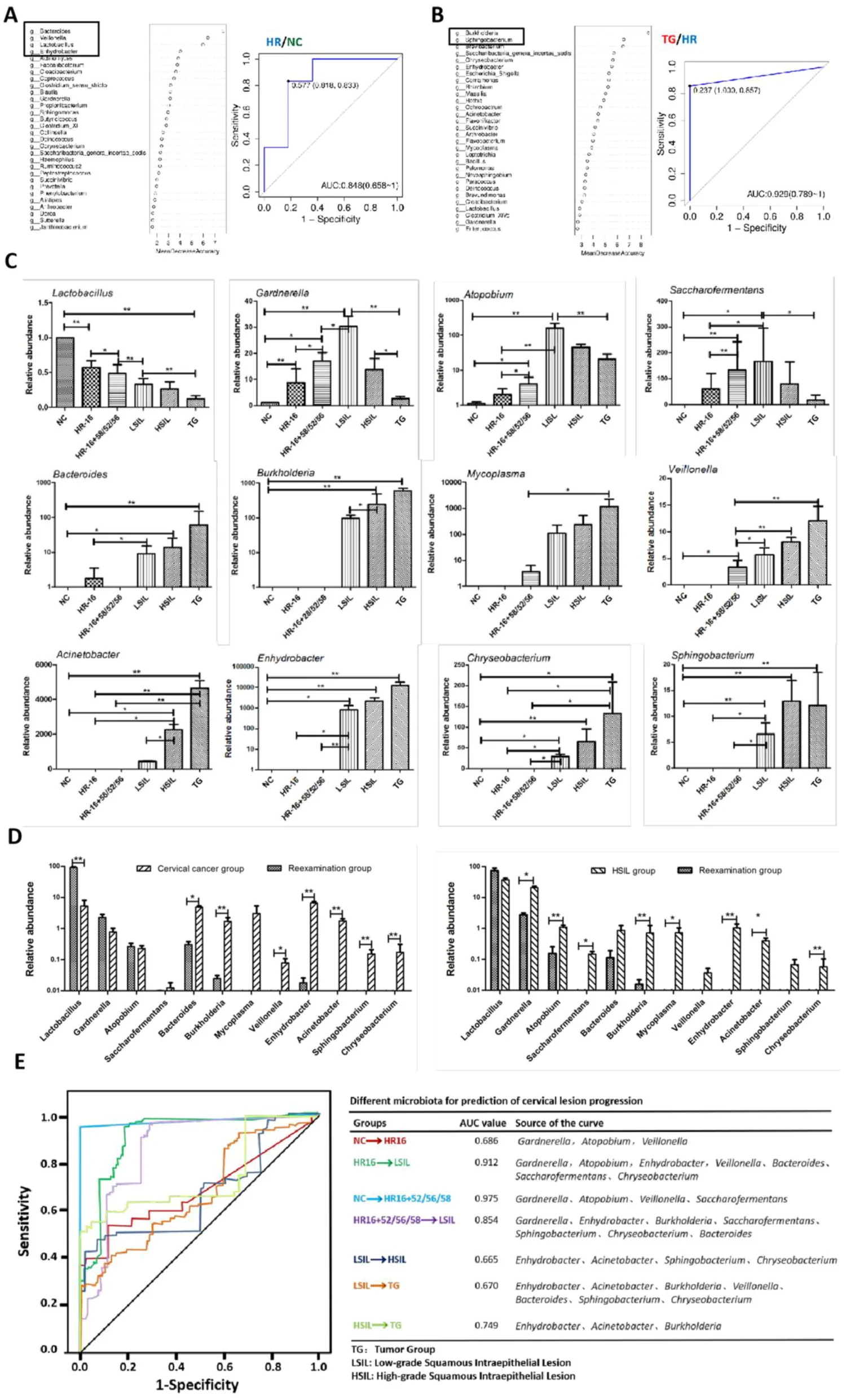
Establishment and verification of prediction biomarkers. (a) and (b) RF predictive model based on the vaginal microbiota profile using the significantly different abundance taxon at the genus level from the Wilcoxon rank-sum test as the input. *p < 0.05, **p < 0.01. (c) Histograms showing the relative abundance of the 12 bio-markers by real-time PCR to confirm the changes in a verification group, including NC, HR-16, HR-16 + 52/56/58, LSIL, HSIL, and TG groups, with a total of 60 samples. *p< 0.05, **p < 0.01. (d) The relative abundance of species (normalized to the abundance of *Lactobacillus* after tumor resection) before and after surgery resection.

**Figure 5.**
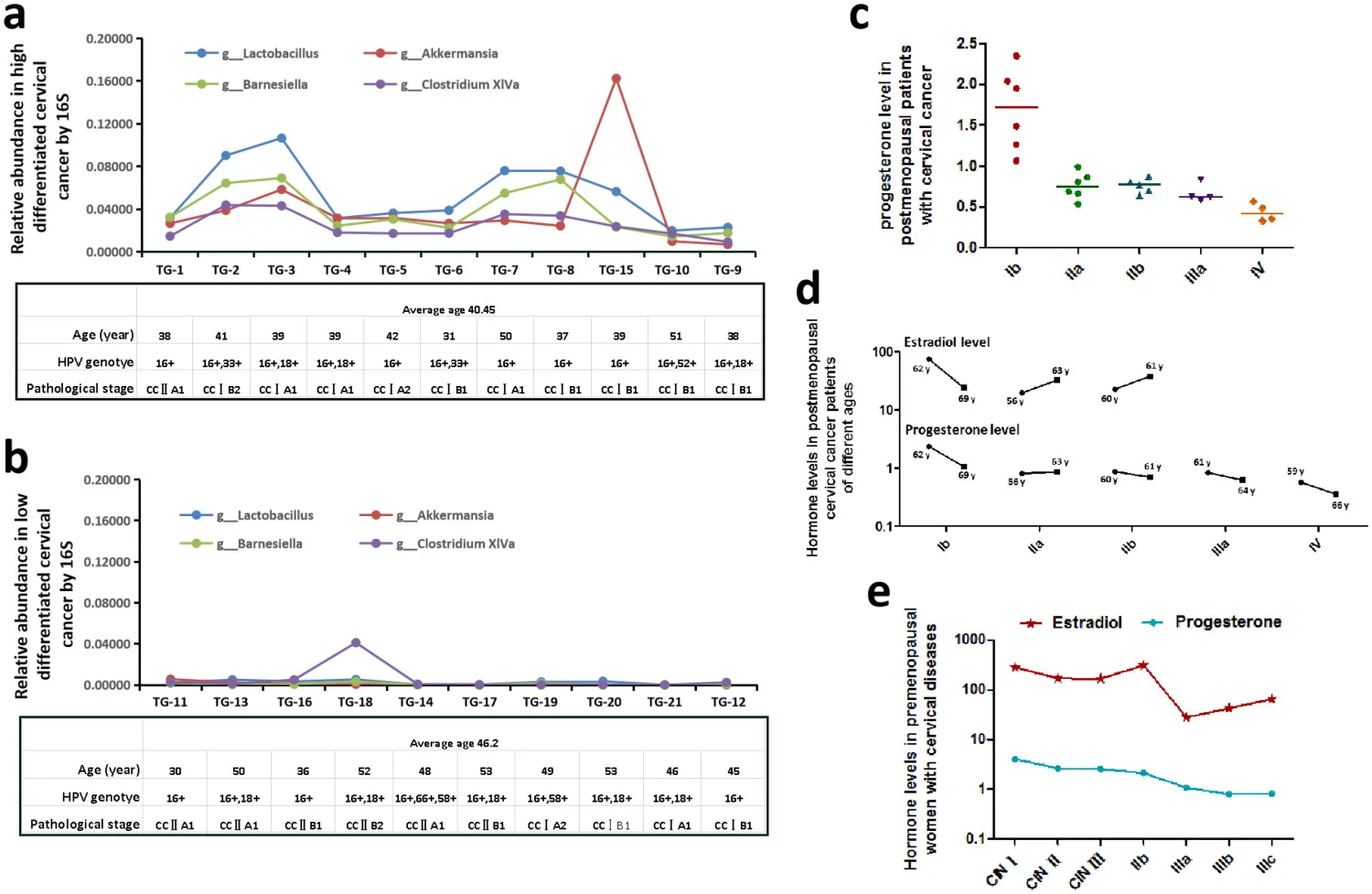
Association between CVM, cervix pathology, and fluctuation in hormone levels. (a–b) Changes of progesterone abundance in cervical cancer. (c-d) Changes of estrogen and progesterone abundance in postmenopausal patients with cervical cancer of different ages. (e) Changes in estrogen and progesterone abundance in premenopausal patients with cervical lesions.

### Cervicovaginal microbiota diversity and composition

To evaluate the overall structure of the cervicovaginal microbiome, a corresponding histogram of species profiling was constructed to visualize the relative abundance of species and the proportion of each group at different classification levels (phylum, class, order, family, and genus). With the development of disease, the relative abundance of *Lactobacillus* gradually decreased, whereas the abundance of many species appeared to increase, including *Firmicutes, Actinobacteria, Proteobacteria, Bacteroidetes, Fusobacteria,* and *Tenericutes* (Figure 2a), consistent with previous studies.

To further explore macrogenomic biomarkers with significant differences, LEfSe analysis (LDA coupled with effect size measurements) was performed, and 190 differentially abundant taxa (4 in NC, 8 in HR16-18, 16 in HR52-56-58, and 162 in TG) among the groups were found, all of which had a log-linear discriminant analysis score (LDA) > 2 and *p* < 0.05 (Figure S5f). A heatmap (top 30) was used to display the differences visually. Figure 2b showed two interesting patterns: *Gardnerella, Atopobium* and *Saccharofermentan* presented an increasing and decreasing trend with the progress of cervical cancer, which was positively associated with the disappearance of the specific infection genotype of HPV52, 56, and 58, while *Escherichia/Shigella*, *Enhydrobacter*, *Bacteroides* and the *Chryseobacterium, Sphingobacterium,* and *Enhydrobacter* were positively correlated with the aggravation of HPV16 and 18 (Figure 2c), while *Gardnerella, Atopobium, Saccharofermentans* disappeared along with the elimination of HPV52, 56, and 58 genotypes, but the definitive reason is still unclear (Figure 2d).

### Functional profile analysis of cervicovaginal microbiota

In addition to identifying alterations in the taxonomic landscape during the progression of cervical cancer, we evaluated the inferred functional composition of the microbiome generated by PICRUSt, which represents hypothesis-generating observations rather than direct measured activity. To determine the interaction between different species during cervical carcinogenesis, the top 20 most abundant species were included for Spearman correlation and SparCC network analysis. Species that increased in the HR group, such as *Gardnerella, Atopobium,* and *Saccharofermentans*, were negatively correlated with *Lactobacillus* in the NC group and increased in the TG group (Figure 3a, Figure S6a).

**Figure 6.**
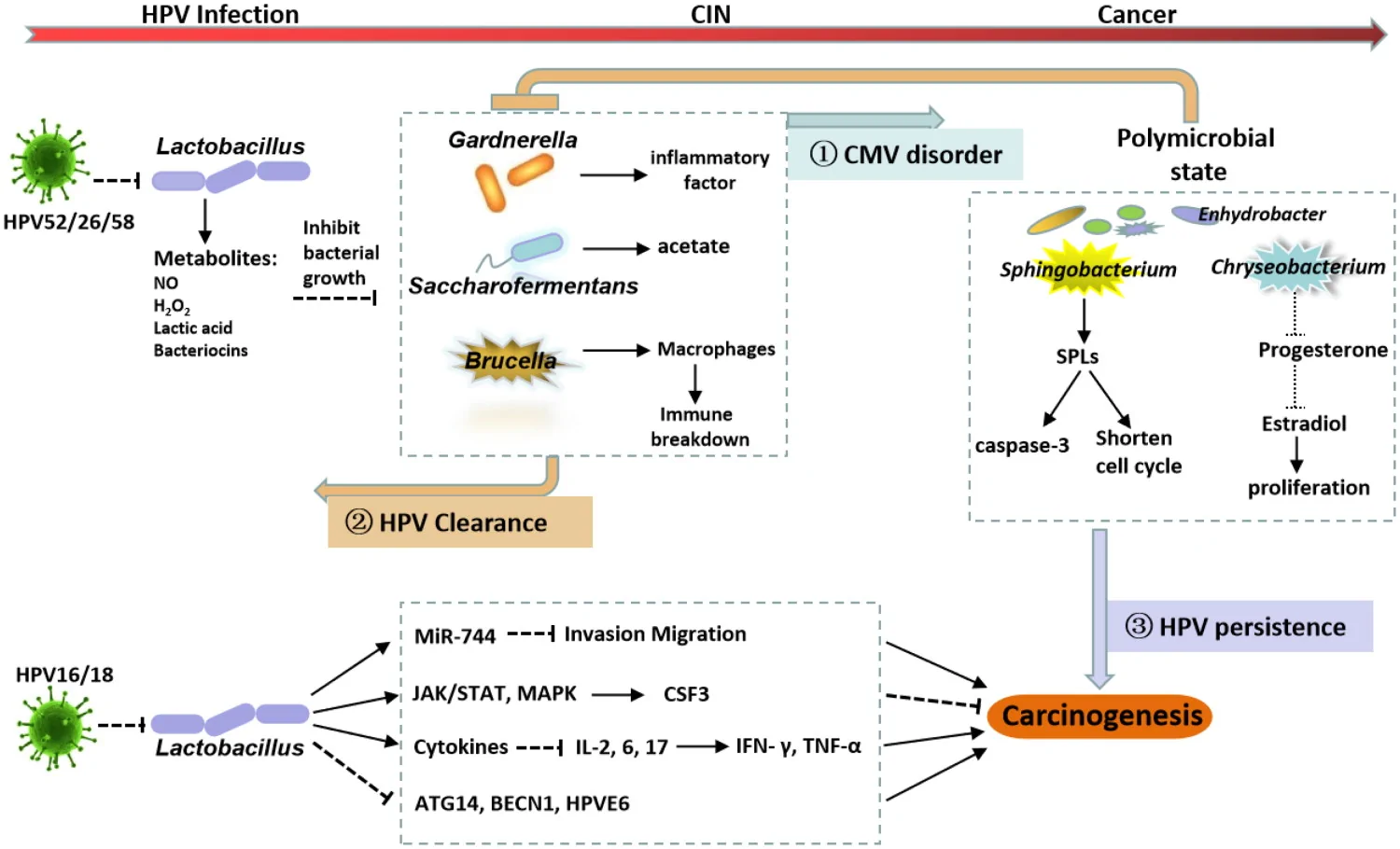
Microbiome and HR-HPV natural history. As the HPV virus enters the basal layer through epithelial wear, CVM gradually transitions from *Lactobacillus*-dominated to obligate anaerobes-dominated, which triggers the inflammatory response and subsequently elevates the microbial diversity. With the progression of cervical cancer, the clearance of HPV52/56/58 may be contributed by the appearance of some species with inhibitory effects on anaerobic bacteria. Additionally, these species further promote HPV16/18 viral persistence and progression by promoting proliferation, invasion, and migration pathways.

To investigate the contributions of microbiome components, Kyoto Encyclopedia of Genes and Genomes (KEGG) pathway analyses were performed based on the predicted functional profiles. A large number of species were found to be significantly enriched in ‘Replication and repair,’ ‘Pathway in cancer,’ and ‘Progesterone-mediated oocyte maturation’ pathways (Figure S6b–d). Specifically, *Lactobacillus* was found to be positively associated with ‘Apoptosis’ and ‘Sphingopid metabolism’ pathways, while *Gardnerella*, *Atopobium,* and *Saccharofermentans* were positively associated with the ‘Bacterial toxins’ pathway, which was negatively associated with cancer progression. Moreover, the increased species, *Enhydrobacter*, *Veillonella*, and *Chryseobacterium*, in the TG group were mainly negatively correlated with ‘Apoptosis’ and positively correlated with ‘Replication and repair,’ ‘metabolism’ and ‘Pathway in cancer,’ which was positively associated with cancer progression (Figure 3b).

Next, we aimed to clarify the association between different species and certain genes involved in specific signaling pathways. Because of the serious host contamination of exfoliated cervical cells and tissues, it was impossible to perform metagenomic sequencing, and the RNA-seq data of HPV-expressing cells were analyzed (Figure S6e). Upon combining with the analysis results of KEGG and RNA-seq, CD99, FN1, AMIGO2, and CYR61 were found associated with ‘Apoptosis’ pathway, and E2F1, CDK1-2, MCM2-7, CDC6-7, CDC25A, RRM1-2, and PCNA mainly participated in ‘cell cycle,’ ‘DNA replication’ and ‘Progesterone-mediated oocyte maturation’ pathways (Table S3). The potential association of changes in specific species on certain genes was considered for further analysis (Figure S6f).

### Identification and verification of potential biomarkers for the cervicovaginal microbiota

The staging of cervical disease is generally determined using a cervical biopsy. Here, we evaluated its progression using a Random Forest (RF) predictive model, which was built using the top species in the importance ranking as biomarkers. From top to bottom, the importance of species in distinguishing between the two groups gradually decreased. The area under the curve (AUC) is defined as the area under the ROC curve; in the case of AUC > 0.5, the closer the AUC is to 1, the better the classification prediction effect. A decrease in *Lactobacillus* and an increase in *Enhydrobacter, Bacteroides*, and *Veillonella* may indicate HPV infection (AUC:0.848; Figure 4a), whereas an increase in *Burkholderia* and *Sphingobacterium* may indicate tumor formation (AUC:0.929; Figure 4b). Overall, the predicted results are consistent with those of the microbial composition analysis.

By combining the results of LEfSe analysis and the RF predictive model, we identified 12 biomarkers. To verify the accuracy, two independent validation cohorts were used: 60 samples covering the entire process of cervical lesions and 13 pairs of samples before and after tumor resection (Table 1). 12 genus-specific primers were synthesized (Figure 4c,d, Figure S7). In general, the validation results were consistent with those of the prediction model. The enrichment of *Enhydrobacter, Sphingobacterium,* and *Chryseobacterium* was consistent with the persistent high prevalence of HPV16/18 across advanced disease stages. In particular, the increase in *Gardnerella, Atopobium,* and *Saccharofermentans* in the LSIL phase decreased or even disappeared in the tumor, which was consistent with the variation trend of HPV52/56/58 (Figure 4e). Four of these species were identified during the progression of cervical cancer.

### Association between cervicovaginal microbiota, cervix pathology, and hormone level fluctuations

Tumors can be divided into four types according to their degree of malignancy: highly differentiated, moderately differentiated, poorly differentiated, and undifferentiated. The higher the degree of differentiation, the lower the degree of malignancy [26]. Among the 21 tumor tissues in the current study, No.1-10 and 15 were poorly differentiated carcinomas, while the remaining 10 specimens were highly differentiated carcinomas. Through data analysis, it was observed that the levels of *Akkermansia, Barnesiella, Clostridium XlV*, and *Lactobacillus* were relatively higher in highly differentiated cancers than in poorly differentiated cancers (Figure 5a,b), indicating that not all recently emerged species in cancer tissue were detrimental. These potential biomarkers may further personalize treatment decisions, thus prolonging the survival of patients with cervical cancer.

Most cervical cancers originate from the cervical squamous cell junction (transformation area), where the immature metaplastic squamous epithelium is metabolically active and highly sensitive to hormones. In this study, we found that progesterone levels in postmenopausal patients with cervical cancer showed a downward trend with the aggravation of the disease course (Figure 5c), which decreased more significantly with increasing age, whereas estrogen levels increased with age in the late stage of cancer (Figure 5d). Furthermore, in premenopausal patients (Figure 5e), progesterone levels continued to decrease gradually with the progression of cancer, whereas estrogen levels showed an increasing trend in the late stage of cervical cancer. However, the relationship between changes in hormone levels and the progression of cervical cancer has not been fully elucidated [27]; the current study found that this pattern may be linked to the presence of *Chryseobacterium,* which has been reported to degrade progesterone [28,29].

## Discussion

In the present study, the cervicovaginal microbiota profile appears to be associated with distinct HPV genotypes across the continuum of cervical carcinogenesis. In cases where HPV 52/56/58 penetrates the basal layer *via* epithelial micro-abrasion, the cervicovaginal microbiota may undergo a gradual shift from a Lactobacillus-dominated state to a community dominated by obligate anaerobes including *Gardnerella*, *Atopobium* and *Saccharofermentans*. This compositional transition may be linked to a local inflammatory response, which may further correlate with subsequent elevation in microbial alpha diversity. With the progression of cervical cancer, newly emerging species (such as *Chryseobacterium*, *Sphingobacterium*, and *Enhydrobacter*) show negative associations with these obligate anaerobes, which may be linked to the declining prevalence of HPV52/56/58 in cancer tissue. These findings are consistent with those of previous reports, indicating that most HPV infections and SIL are transient. While HPV 16/18 enters the basal layer, CVM diversity may become more complex and dysbiotic, with the previously dominant *Gardnerella*, *Atopobium* and *Saccharofermentans* no longer occupying the leading taxonomic positions. This microbial community rearrangement may correlate with other co-existing microbiota features, and may be associated with the sustained high prevalence of HPV 16/18 throughout advanced disease stages and malignant progression, potentially *via* pathways related to altered macrophage proliferation, invasion and migration (Figure 6).

We note that while the aforementioned genera, including *Chryseobacterium, Sphingobacterium*, and *Enhydrobacter*, are not traditionally recognized as dominant members of the healthy cervicovaginal microbiota, a growing body of studies has documented their detectable presence in cervical specimens. We therefore discuss the plausible scenario that these taxa may represent low-abundance yet biologically relevant residents of the microbial community, rather than trivial technical artifacts. Furthermore, rigorous quality-control procedures were enforced across the entire experimental workflow: (1) blank DNA extraction controls and no-template PCR controls were included in every batch of sample processing, sequenced in parallel with experimental samples, and subsequently applied to filter out potential contaminant taxa; (2) all samples with total 16S rRNA gene reads below our predefined threshold were excluded at the initial quality-filtering stage; (3) additional sensitivity analyses were performed by removing all taxa commonly reported as laboratory contaminants from the dataset, which confirmed that the association signals for these three genera remained statistically significant after this adjustment. We also explicitly acknowledge that residual low-biomass contamination cannot be entirely ruled out, and recommend that future studies with larger independent cohorts further validate the potential of these taxa as candidate biomarkers for cervical disease.

*Chryseobacterium*, a specific microbial species was associated with fluctuations in hormone levels. Hormonal fluctuations are involved in the distortion of the immune system and the promotion of cancer. Recently, certain components of the intestinal microbiota, such as *Gammaproteobacteria, Myxococcales*, and *Klebsiella aerogenes*, were identified to have a positive association with estradiol [30], while others, such as *Prevotellaceae, Slackia*, and *Butyricimonas,* have a negative associations [31,32]. However, little is known about the relationship between hormonal fluctuations and specific microbial species in the cervicovaginal microbiome and the interplay between them. Notably, the abundance of *Chryseobacterium* gradually increases with the progression of cervical lesions, especially in cancer tissues. *Chryseobacterium* is a rare human pathogen that can cause serious infections, especially in individuals with compromised immune systems [33–35]. *Chryseobacterium* can secrete active metalloproteinases (MMP) [36], and an increase in MMP is associated with tumor invasion and metastasis [37]. In addition, *Chryseobacterium* is reported to degrade progesterone [28], which inhibits estradiol proliferation in *HeLa* cells [38]. However, the expression level of estradiol in cervical cancer is significantly increased [27,39]. This suggests that *Chryseobacterium* may be linked to higher estradiol activity and increased the proliferation of cervical cancer cells *via* progesterone degradation, though this hypothesis requires further functional validation.

*Sphingobacterium* has been identified in a number of patients with compromised immunity, such as those with liver transplants, rheumatoid arthritis, and chronic kidney disease patients [40–47]. S*phingobacterium* contain sphinophydropids (SPLs) and ceramides. The former can activate macrophages, induce an innate immune response, and promote bacterial clearance, which can induce DNA fragmentation, activate caspase-3, and shorten the cell cycle [28]. In conclusion, the inflammatory response caused by *Sphingobacterium* may be linked to the occurrence and development of cervical cancer. We also observed a positive association of *Enhydrobacter* with cervical lesions [48]. As a component of the skin microbiome [48], *Enhydrobacter* has been reported to participate in the development of conditions such as atopic eczema and psoriasis. However, its role in the CVM remains to be elucidated, and no additional reports are available for comparison.

In addition, *Gardnerella, Atopobium,* and *Saccharofermentans* were found to be potentially associated with HR-HPV infections. It seems that the presence of symbiotic bacteria (such as *Lactobacillus iners*) with the ability to stimulate local immune responses may be essential for the elimination of HPV infection [49], while the absence of *Lactobacillus crispatus* alongside *Atopobium* and *Gardnerella* could serve as a defining factor for severe cervical lesions [50,51]. However, the roles of these microorganisms remain unclear and require further investigation. Our study found a core microbiota consisting of *Gardnerella* and *Atopobium* as the two dominant phyla in the HPV-infected group, which is consistent with previous reports. *Gardnerella*, as mentioned above, is regarded as a risk factor for the disruption of CVM homeostasis. It has been reported that *Gardnerella* can accelerate vaginal epithelial cell death by regulating CD59, which increases vaginolysin (VLY), thus reducing *Lactobacillus* colonization [52]. Another anaerobic bacterium, *Atopobium*, has been proposed as a molecular marker that may contribute to virus persistence [43].

Furthermore, we noticed an increased abundance of *Saccharofermentans* in patients with HPV infection. Interestingly, *Saccharofermentans* are known to produce acid-enhancing metabolites that lower the pH and cause the death of pathogenic bacteria, as indicated by the negative correlation in the network. In addition, *Saccharofermentans* were found in the vagina of adult macaques [53], and the main end-product was acetate. Previous studies have revealed that glycogen and *Lactobacillus* are less abundant in the vaginal environment of macaques [54], whereas the main acid constituent is acetate. These results were consistent with ours in that *Lactobacillus* content gradually decreased, whereas the species responsible for producing acetic acid (*Saccharofermentans*) increased during the progression of cervical lesions. Therefore, our results suggest that monitoring the presence of *Gardnerella, Saccharofermentans,* and subsequently elevated microbes can be used as a marker to identify persistent HR-HPV infection with progression risk.

Moreover, distinct microbial differences (including *Akkermansia, Barnesiella, Clostridium XlV*, and *Lactobacillus*) were observed as the cervical cancer progressed to advanced FIGO stages. The microbiota contributes to cancer not only by the procarcinogenic roles played by certain pathogens but also *via* the influence of their metabolome. Colonization of *Akkermansia muciniphila* has been reported to promote mucosal wound healing and induce adaptive intestinal immune responses during homeostasis [55]. Genera *Barnesiella* was related to immune regulatory cells, and its presence suggests that the intestinal environment is not prone to inflammation [56]. *Clostridium XIVa* has a strong role in inducing important anti-inflammatory molecules such as IL-10 and enhancing T_reg_ cell abundance [57]. However, the mechanisms by which these genera mediate these effects remain poorly understood and have not been identified in CVM. The results of this study improve our understanding of the role of microbes as cervical cancer progresses to advanced FIGO stages.

The cervico-vaginal microbiota is known to be influenced by numerous host-related factors, including age, menopausal status, hormonal background, smoking, contraceptive use, and sexual behavior. To clarify the association between cervicovaginal microbiota dysbiosis, persistent HPV infection and cervical lesion progression, this study implemented systematic control over the aforementioned potential confounding factors during both the study design phase and subsequent statistical analysis phase, to minimize non-target interference. (1) Variables directly controlled by uniform exclusion criteria: In the design phase of this study, key confounding variables were pre-controlled through strict inclusion and exclusion criteria, such as excluding individuals with a history of HPV vaccination, excluding pregnant subjects and those within 8 weeks postpartum, and specifying requirements for abstinence and antibiotic prohibition before sampling. (2) Core host factors systematically included in statistical analysis: we further incorporated age, menopausal status, smoking history and contraceptive use as covariates to correct their potential confounding effects. In addition, this study established a serum sub-cohort consisting of 21 premenopausal and 25 postmenopausal cervical cancer patients (age range 54–69 years), which provides a basis for subsequent exploration of the shaping effect of hormonal background on the microbiota across different menopausal stages.

### Study limitations

Several limitations should be explicitly acknowledged: limited sample sizes in some genotype-specific subgroups reduce statistical power to detect subtle microbial associations, and our hormonal analyses remain exploratory due to small sample size. Even though our validation cohort provided consistent support for the hypothesized links between cervical disease states and microbiota alterations, its relatively modest size imposes an additional methodological constraint. Nevertheless, the current findings further strengthen the plausibility of microbiota modulation as a viable intervention to modify cervical disease progression. While the findings generated in this work are meaningful and warrant further in-depth exploration, the present investigation is inherently observational in nature. The biological mechanisms proposed in this study remain speculative at this stage, independent larger cohorts and follow-up experimental studies are therefore required to rigorously validate these hypothesized causal links.

## Supplementary Material

Supplementary tables and figures.docx

## Data Availability

The Supplementary Material for this article are included in the supplementary material. All datasets generated for this study are shared openly in a data repository https://www.ncbi.nlm.nih.gov/sra/PRJNA1403155.
