# Supplementary material for: Cervicovaginal microbiome modulates HPV genotype-specific clearance/persistence: role of bacterial dysbiosis in cervical carcinogenesis
Source: Ann Med. 2026 Nov 6;58(1):2754577. doi: 10.1080/07853890.2026.2754577 (PMC13757928; doi:10.1080/07853890.2026.2754577)
Supplement: Supplementary file 1 [file IANN_A_2754577_SM6916.docx]

**Supplementary Materials**

**Supplementa Table 1**

**The prevalence rates of 24 HPV genotypes**

| **Total number of samples (n=24641，22.50%)** | | | | | |
| --- | --- | --- | --- | --- | --- |
| **Single infection (n=4421)** | | | **Multiple infection (n=1124)** | | |
| **Genotype** | **Positive number** | **Positive rate** | **Genotype** | **Positive number** | **Positive rate** |
| **16+** | 728 | 16.47% | **16+** | 326 | 29.00% |
| **58+** | 536 | 12.12% | **58+** | 226 | 20.11% |
| **52+** | 343 | 7.76% | **52+** | 192 | 17.08% |
| **56+** | 256 | 5.79% | **56+** | 186 | 16.55% |
| **51+** | 228 | 5.16% | **18+** | 157 | 13.97% |
| **18+** | 219 | 4.95% | **42+** | 130 | 11.57% |
| **43+** | 214 | 4.84% | **66+** | 127 | 11.30% |
| **11+** | 201 | 4.55% | **11+** | 126 | 11.21% |
| **66+** | 201 | 4.55% | **6+** | 122 | 10.85% |
| **53+** | 187 | 4.23% | **51+** | 115 | 10.23% |
| **68+** | 177 | 4.00% | **68+** | 109 | 9.70% |
| **33+** | 174 | 3.94% | **33+** | 102 | 9.07% |
| **6+** | 157 | 3.55% | **43+** | 89 | 7.92% |
| **42+** | 146 | 3.30% | **53+** | 84 | 7.47% |
| **39+** | 128 | 2.90% | **35+** | 81 | 7.21% |
| **31+** | 113 | 2.56% | **59+** | 77 | 6.85% |
| **35+** | 105 | 2.38% | **39+** | 71 | 6.32% |
| **59+** | 101 | 2.28% | **31+** | 65 | 5.78% |
| **84+** | 67 | 1.52% | **45+** | 36 | 3.20% |
| **45+** | 66 | 1.49% | **84+** | 28 | 2.49% |
| **44+** | 30 | 0.68% | **73+** | 16 | 1.42% |
| **73+** | 23 | 0.52% | **82+** | 10 | 0.89% |
| **82+** | 17 | 0.38% | **26+** | 8 | 0.71% |
| **26+** | 4 | 0.09% | **44+** | 8 | 0.71% |

**Red presents the high-risk genotype of HPV (HR-HPV)**

**Blue presents the low-risk genotype of HPV (LR-HPV)**

**Supplemental Table 2**

**The infection rate of HR-HPV in various cervical lesions**

| **HPV Genotype** | **HPV infection (n=5545)** | | **LSIL (n=104)** | | **HSIL (n=78)** | | **CC (n=77)** | |
| --- | --- | --- | --- | --- | --- | --- | --- | --- |
|  | **Positive number** | **Positive rate** | **Positive number** | **Positive rate** | **Positive number** | **Positive rate** | **Positive number** | **Positive rate** |
| **16** | 1054 | 19.01% | 33 | 31.73% | 38 | 48.72% | 77 | 100% |
| **58** | 762 | 13.74% | 21 | 20.19% | 13 | 16.67% | 11 | 14.28% |
| **52** | 535 | 9.65% | 24 | 23.07% | 15 | 19.23% | 7 | 9.09% |
| **56** | 442 | 7.97% | 11 | 10.89% | 4 | 5.13% | 0 | 0 |
| **18** | 376 | 6.78% | 9 | 8.65% | 13 | 16.67% | 29 | 37.66% |
| **16+58/52/56** | 149/326 | 45.70% | 19/33 | 57.57% | 15/38 | 39.47% | 18/77 | 23.37% |
| **18+58/52/56** | 34/157 | 21.65% | 2/9 | 22.22% | 1/13 | 7.69% | - | - |
| **16+18** | 34/326 | 10.42% | 5/33 | 15.15% | 8/38 | 21.05% | 29/77 | 37.66% |

| **Supplenmental Table 3**  **Up-regulated and Down-regulated genes in HPV expressing NIKS cells** | |
| --- | --- |
| **pathways** | **Up-regulated genes in HPV expressing cells** |
| **Cell cycle** | E2F1, CDC7, CDK1, CDC6, RBL1, TTK, MCM2, MCM3, MCM4, CDK2, CDC25A, MCM5, MCM6, CCNE2, CDC45, CDKN2A, MCM7, PCNA, MDM2, CCNA2 |
| **DNA replication** | POLE, POLA1, MCM2, MCM3, MCM4, MCM5, MCM6, RPA1, RFC5, POLD3, PRIM1, RPA2, RFC3, MCM7, PCNA |
| **Pathways in cancer** | E2F1, CCNE2, MSH6, CDKN2A, MSH2, MDM2, BRCA2, BIRC3, CDK2 |
| **Mismatch repair** | POLD3, RFC5, RPA1, MSH6, RPA2, RFC3, MSH2, PCNA |
| **Nucleotide excision repair** | POLD3, RFC5, RPA1, RPA2, RFC3, POLE, PCNA |
| **Homologous recombination** | POLD3, RPA1, RPA2, MRE11A, BRCA2, RAD54B |
| **p53 signaling pathway** | CCNE2, CDK1, CDKN2A, RRM2, MDM2, CDK2 |
| **Pyrimidine metabolism** | POLD3, PRIM1, RRM2, RRM1, POLE, POLA1 |
| **Purine metabolism** | POLD3, PRIM1, RRM2, RRM1, POLE, POLA1 |
| **Progesterone-mediated oocyte maturation** | CDK1, MAPK13, CCNA2, CDC25A, CDK2 |
| **Base excision repair** | POLD3, POLE, PCNA |
|  |  |
| **pathways** | **Down-regulated genes in HPV expressing cells** |
| **Regulation of catabolic process** | MAGED1, PTHLH, LEPRE1, CYP27B1, APOE, PDGFA, IGFBP6, JAG1, SPARC, COMT, EMP3, PLAU, TIMP1 |
| **Regulation of cell growth** | MAGED1, TNFRSF6B, TNFRSF21, PEG10, RHOB, PNPLA6, TRADD, ERCC2 |
| **Cell death** | MAGED1, TNFRSF6B, TNFRSF21, PEG10, RHOB, PNPLA6, TRADD, ERCC2 |
| **Induction of apoptosis** | AMIGO2, COL7A1, TNC, ITGB4, RHOB, CD99, LOXL2, SDC3, CYR61, FN1 |
| **Induction of programmed cell death** | AMIGO2, COL7A1, TNC, ITGB4, RHOB, CD99, LOXL2, SDC3, CYR61, FN1 |
| **Positive regulation of nucleotide metabolic process** | TYMP, PDGFA, RHOB, JAG1, PLAU, PNPLA6, CYR61 |
| **Positive regulation of cyclic nucleotide metabolic process** | KCNN4, GYLTL1B, APOE, MT2A, GAA, SH3BGRL3, ERCC2 |
| **Positive regulation of cyclic nucleotide biosynthetic process** | TYMP, PDGFA, APOE, RHOB, JAG1, PLAU, PNPLA6, CYR61 |
| **Negative regulation of lipid biosynthetic process** | TYMP, PDGFA, APOE, RHOB, JAG1, PLAU, PNPLA6, CYR61 |
| **Growth** | PTHLH, CYP27B1, FHL2, SPARC, EXT1, ERCC2 |
| **Positive regulation of nucleotide biosynthetic process** | PTHLH, CYP27B1, FHL2, SPARC, EXT1, ERCC2 |
| **Aminoglycan metabolic process** | TNC, UTRN, SERPINH1, CYR61, ERCC2 |
| **Positive regulation of epidermis development** | SERPINE2, PDGFA, APOE, RRAS, JAG1 |
| **Regulation of response to external stimulus** | CYP27B1, ECH1, APOE, PNPLA6, PLBD2 |
| **Chemotaxis** | SERPINE2, PDGFA, APOE, RRAS, JAG1 |

**Figure S1. HPV distribution in China.** 1.7 million Chinese woman samples identified from PubMed databases screening were analysed.

**Figure S2: Study cohort.** Analysis of 1.7 million Chinese woman samples identified from PubMed databases screening. 24641 women in Shandong Province were recruited for the HPV test. 228 samples were identified by 16S rRNA sequencing, including negative control group, tumor group, and 18 different HPV positive specimens in the Chinese population. 60 additional samples and a longitudinal cohort of samples before and after tumor resection (HSIL, 6 pairs; TG, 7 pairs) for validation study. 46 serum samples were used for hormone detection.

**Figure S3. Comparison of bacterial richness, evenness and diversity in each genotype of HPV infection specimens.** (a) Comparative taxonomic profile of different HPV genotypes. The genera with significant richness difference (P <0.05, computed by STAMP) between the two groups are shown. (b) OTU number in HPV single genotype infection and multiple genotype infection. (c) OTU Venn diagram analysis and the number of OTUs shared between groups and unique within groups in each HPV infection genotypes.

**Figure S4. Alpha diversity and diversity analysis based on genera within or between groups in NC, HPV16, 18, 52, 56, 58 and TG groups.** (a) Shannon diversity index in HPV single genotype infection. (b) ANOSIM analysis in HPV single genotype infection. (c) and (d) The difference in the abundance of CVM communities at the Phylum, Genus level respectively. (e) MRPP analysis.

**Figure S5. Composition of microbial communities in NC, HR16-18, HR52-56-58 and TG groups.** (a) Shannon diversity index. (b)MRPP analysis. (c) The top 20 species richness was analyzed by species classification tree. Different colors indicate different taxonomic levels, the size of the circle represents the relative abundance of the taxonomic level, and the number below the taxonomic name represents the relative abundance percentage. (d)-(e) Boxplots with relative abundance of the different microbial taxa at genes and all levels. (f) LEfSe analysis (LDA coupled with effect size measurements) was performed, which emphasized the statistical significance and biological correlation; All of the showed taxon had a log Linear discriminant analysis score（LDA）>2 and p<0.05.

**Figure S6. Functional analysis of CVM during cervical carcinogenesis.** (a) Spearman correlation coefficient analysis of dominant species through the CORRPLOT package of R software. (b) Enrichment analysis of differential gene of of cell cycle pathway. (c) Enrichment analysis of differential gene of of signaling pathway. (d) Enrichment analysis of differential gene of of metabolism pathway. (e) RNA-seq analysis. KEGG annotations with significant abundance differences (P < 0.05) are shown. (f) The effect of changes in specific species on certain genes

**Figure S7.** Real-time PCR results of Sphingobacterium, Chryseobacterium and Saccharofermentans with agarose gel electrophoresis.
